# The HTLV-1 encoded bZIP factor promotes cell proliferation and genetic instability through activation of oncogenic microRNAs

**DOI:** 10.1186/1742-4690-11-S1-O43

**Published:** 2014-01-07

**Authors:** Céline Vernin, Christiane Pinatel, Antoine Gessain, Olivier Gout, Marie-Hélène Delfau-Larue, Nicolas Nazaret, Catherine Legras-Lachuer, Eric Wattel, Franck Mortreux

**Affiliations:** 1 Université de Lyon 1, CNRS UMR5239, Oncovirologie et Biothérapies, Laboratoire de Biologie Moléculaire de la Cellule, Faculté de Médecine Lyon Sud, Pierre Bénite, France; 2 Université Lyon I, Faculté de Médecine et de Pharmacie de Lyon, ISPBL ProfileXpert-LCMT, Lyon, France; 3 Institut Pasteur, Unité d'Epidémiologie et Physiopathologie des Virus Oncogènes, Paris, France; 4 Fondation Rothschild, Service de Neurologie, Paris, France; 5 CHU Henri Mondor, Laboratoire d'Immunologie, Créteil, France; 6 Centre de Recherche sur le Cancer de Lyon, Centre Léon Bérard, Lyon, France; 7 Université Lyon I, Service d'Hématologie, Pavillon Marcel Bérard, Centre Hospitalier Lyon-Sud, Pierre Bénite, France

## 

Viruses disrupt their host cells microRNAs (miRNAs) network for facilitating their replication. That of HTLV-1 relies on the clonal expansion of its host CD4+ and CD8+ T-cells yet the virus causes adult T-cell leukemia/lymphoma (ATLL) that is regularly of the CD4+ phenotype. Infected cells express Tax and HBZ viral oncoproteins. Tax is expressed in untransformed cells where it promotes cell proliferation, genetic instability and miRNAs deregulation whereas in contrast, HBZ is expressed by untransformed and malignant T-cells where hitherto, it is considered to promote cell proliferation and to silence virus expression. Here we show that an HBZ/miRNAs axis promotes cell proliferation and genetic instability. Infected CD4+ but not CD8+ T-cells were found to overexpress oncogenic miRNAs such as miR-17 and miR-21. HBZ activated these miRNAs via a posttranscriptional mechanism while in addition to promoting cellular growth; HBZ decreased DNA stability. These effects were alleviated by either miR-21/miR-17 knockdown or by the ectopic expression of OBFC2A, a factor that protects genome stability and that we found targeted by miR-17 and miR-21 in HTLV-1 infected CD4+ T-cells. This considerably extends the oncogenic potential of HBZ and suggests that viral expression might be involved in the remarkable genetic instability of ATLL cells.

